# A novel 1p33p32.2 deletion involving SCP2, ORC1, and DAB1 genes in a patient with craniofacial dysplasia, short stature, developmental delay, and leukoencephalopathy: A case report

**DOI:** 10.1097/MD.0000000000023033

**Published:** 2020-11-06

**Authors:** Maoying Jiang, Shanlin Wang, Fei Li, Juan Geng, Yiting Ji, Ke Li, Xiaodong Jiang

**Affiliations:** a Hangzhou Children's Hospital, Behavioral Pediatric Department &Child Primary Care Department, Hangzhou; b Developmental and Behavioral Pediatric Department & Child Primary Care Department, MOE-Shanghai Key Lab for Children's Enviromental Health, Xinhua Hospital Affiliated to Shanghai Jiaotong University School of Medicine, Shanghai; c Hangzhou Joingenome Diagnostics, Hangzhou; d Developmental and Behavioral Pediatric Department, Shanghai Children's Hospital, Affiliated To Shanghai Jiaotong University School of Medicine& MOE-Shanghai Key Lab for Children's Environmental Health, Shanghai, China.

**Keywords:** 1p33p32.2 deletion, chromosome deletion, microarray analysis, multiple abnormalities, *ORC1*, *SCP2*

## Abstract

**Introduction:**

Microdeletion syndromes occur from deletion of 5Mb of a chromosome in approximately 5% of patients with unexplained intellectual disability. Interstitial microdeletions at bands 1p33 and 1p32.2 of the short arm of chromosome 1 are rare and have not been previously reported in relation to disease.

**Patient concerns:**

We present a case of a 39-month boy with Pierre Robin sequence, development delay/intellectual disability, growth retardation, short stature, leukoencephalopathy, craniofacial dysplasia, and speech delay. The child was referred to the Child health care department in October 2014 for his delayed language development and aggravated aggression.

**Diagnosis:**

Molecular diagnostic testing with G-band karyotyping was normal but clinical microarray analysis detected a 10 Mb microdeletion at 1p33p32.2.

**Interventions:**

The patient received rehabilitation.

**Outcomes:**

Three candidate genes were pinpointed to the deleted area, including *ORC1, SCP2,* and *DAB1*. Phenotype-genotype analysis suggested that these three genes are likely to be responsible for the main phenotypes observed in the patient, such as microcephaly, growth retardation, short stature, leukoencephalopathy, and development delay/intellectual disability.

**Conclusions:**

The spectrum of phenotypes this case presented with are likely to be caused by 1p33p32.2 deletion which could represent a new microdeletion syndrome.

## 1 Introduction

Microdeletion syndromes occur in approximately 5% of patients with unexplained intellectual disability.^[1]^ Interstitial deletions of chromosomes are rare events, but more than one hundred microdeletion syndromes have been reported in the Online Mendelian Inheritance in Man (OMIM) database.^[2]^ The common phenotypes of microdeletion syndromes include congenital anomalies, intellectual disability, autism, epilepsy, and neuropsychiatric disorders.^[3]^ So, karyotyping, multiple ligation-dependent probe amplification, fluorescent in situ hybridization are now routinely utilized for genetic testing of patients with intellectual disability, multiple malformations, unidentified syndromes, and chromosome diseases.^[4]^

Microdeletions can be hard to detect using traditional methods because of their small size, usually less than 5Mb. Chromosomal microarray analysis (CMA), has helped define the size of copy number variations (CNV) and their gene content, and has promoted novel disease gene discoveries and genotype-phenotype correlation studies.^[5,6]^ The diagnostic yield of CMA testing ranges from 15% to 20% for individuals with unexplained developmental delay/intellectual disability (DD/ID), autism spectrum disorder, or multiple congenital anomalies.^[7]^ The American College of Medical Genetics and Genomics, Italian Society of Human Genetics, and Canadian College of Medical Geneticists^[8]^ all recommend the use of CMA as the first-tier diagnostic test for individuals with developmental disabilities or congenital anomalies.^[9]^

Here, we report on a case of a 39-month-old patient with Pierre Robin sequence, DD/ID, growth retardation, short stature, myelination delay of white matter, craniofacial deformity and speech delay. Molecular diagnostic testing with G-band karyotyping was normal but CMA detected a 1p33p32.2 deletion. The deletion was 10Mb in size, which may be the largest one described so far in this region.

## 2 Case presentation

The 39-month-old male patient was the second child of non-consanguineous parents. The patient had a 20-year-old healthy sister in college. His mother became pregnant at 42 years old, and was screened, because the pregnancy was high risk, 4 months before delivery. She had symptoms of transient anxiety in the first trimester and she had a history of one miscarriage due to unknown cause. The infant was delivered by cesarean section (size 50 cm, weight 3,030 g, Apgar 7/10/10, amniotic fluid III pollution) following a normal pregnancy. He was transferred to the local hospital because of dyspnea and groaning after delivery. On physical examination at birth, Pierre Robin sequence was suspected as he presented with micrognathia, tenesmus of the rear of the tongue and high palate. An echocardiogram indicated a newborn murmur was secondary to a patent ductus arteriosus and atrial septal defect which resolved spontaneously at 5 months of age and was confirmed by doppler echocardiography. The pediatric patient suffered from hearing disorder, and failed the newborn hearing screening test (Automated Auditory Brainstem Response test) at the 4^th^ day of birth. He required outpatient care because of difficulty in feeding since the neonatal period. At one year of age he developed tonic-clonic seizure disorder when crying, but the electroencephalogram was normal. The patient presented with growth retardation and language delay at 18 months.

**Figure 1:**
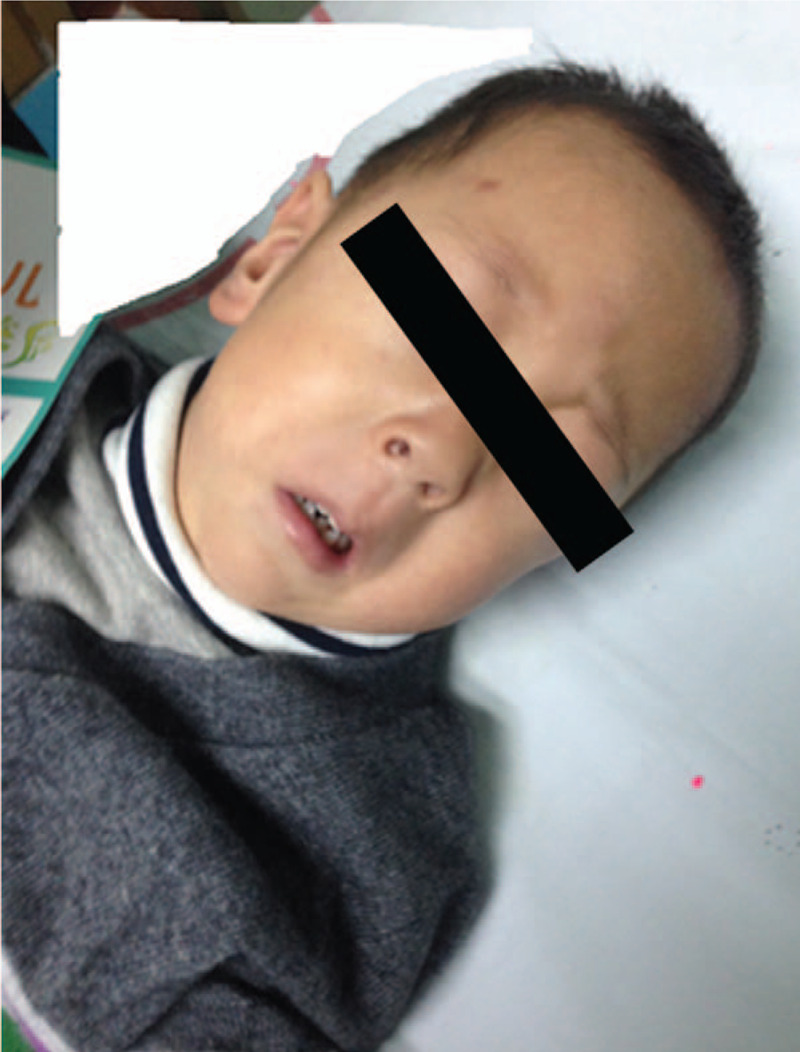
Photograph showing the facial characteristics of the 39-month-old boy presented in this case study. He presented with a long face with micrognathia, frontal bossing, sparse eyebrows, prominent philtrum, abnormal teeth (dental crowding), high palate, squint and nystagmus.

At 39-months old, the patient was referred to Child health care department in October 2014 for his language development and aggravated aggression. On examination, the patient had a height of 89.5 cm (<-2SD), weight of 10.5 kg (<-3SD), head circumference of 45.5 cm (<4SD), which were significantly lower than the normal range for 39-month-old children. He presented with a long face with micrognathia, frontal bossing, sparse eyebrows, prominent philtrum, abnormal teeth (dental crowding), high palate, squint and nystagmus (Fig. 1). The patient learnt to lift his head at 9 months, sit on his own at 11 months, stood on his own at 16 months, walked by himself at 18 months, and learnt some simple pronunciation at 20 months. The patient could only express a few words about their basic needs at their admission examination. The patient was examined by the Autism Diagnostic Observation Schedule and considered to have no autism. Hearing screening was normal. The patient showed aggressive behavior, such as hitting and biting with gradual increase in degree and frequency. The levels of total and low-density lipoprotein (LDL) serum cholesterol, routine chemistry, thyroid function, serum urate, creatine kinase, and blood metabolic screening was normal. Magnetic resonance imaging was performed, which showed abnormal myelination of the white matter, demonstrated as bilateral non-hypointense signals in the occipital lobe and anterior limb of the internal capsule (leukoencephalopathy). Allergen examination showed that he was allergic to some food such as mushroom, codfish and wheat. The Gesell developmental diagnosis scale showed scores far below the normal level which suggested a global development delay. Karyotype analysis and CMA analysis revealed his parents were unrelated and had normal chromosomes.

Peripheral blood lymphocytes were collected from the patient and were cultured in RPMI media augmented with 10% fetal calf serum. Twenty metaphase cell chromosomes were analyzed for abnormalities by a standard technique.^[10]^ Karyotyping was performed in 5 metaphases by a routine procedure.

Genomic DNA was extracted from peripheral blood collected from the patient and his parents. A chromosomal microarray analysis was performed with a CytoScan HD system (Affymetrix, USA) in accordance with the manufacturer's instruction. The CytoScan HD array is characterized with > 2,600,000 probes including 750,000 genotype-able single nucleotide polymorphism probes and > 1,900,000 CNV probes. All data was visualized and analyzed with the Chromosome Analysis Suite (ChAS) software Package (Affymetrix). The CNV calling threshold was set at 25 consecutive probes encompassing 50 kb or more in length.

**Figure 2:**
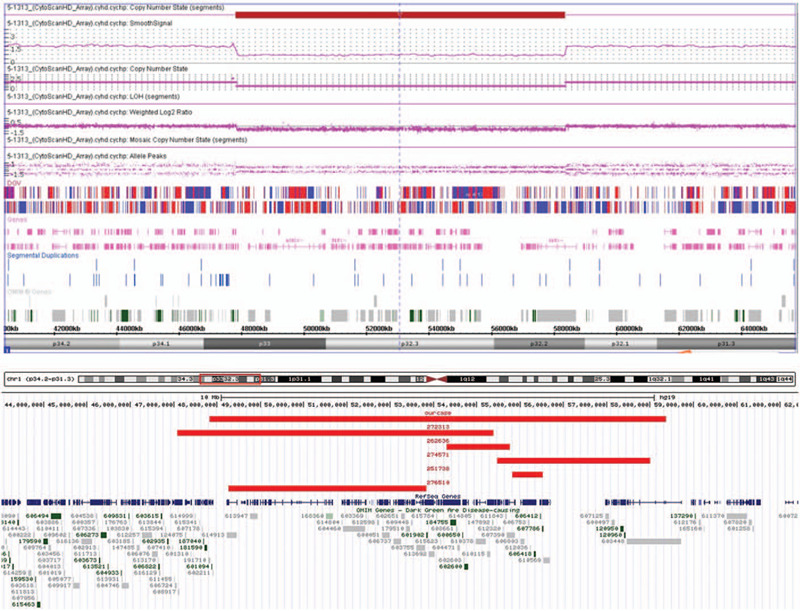
Affymetrix CytoScan HD array analysis including weighted log2 ratio, copy number state, mosaic copy number state, and allele difference for chromosome 1 showed an interstitial deletion at 1p33-p32.2. The genomic coordinates (hg19) are chr1: 47831383-58364913. The deleted region is denoted by a red bar.

**Figure 3:**
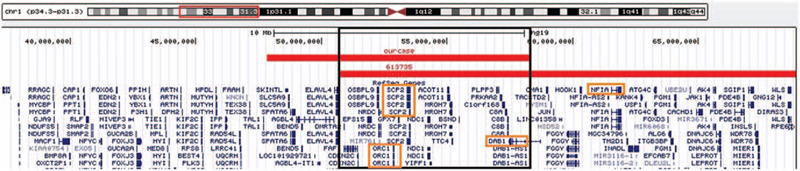
Genotype comparison between our patient and 1p31.3p32.2 deletion syndrome. The *NFIA* gene was not involved in our case. The orange box is the possible pathogenic gene area. The black box represents the area of overlapping deletion with the other two reported cases.

**Figure 4:**
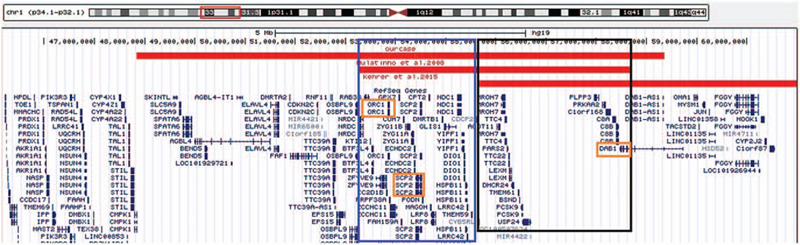
Genotype comparison between our patient and 1p32 deletion. The *DAB1* gene might be a candidate gene for some of the phenotypes seen in our case. The black and blue box represent the area of overlapping deletion with the other two reported cases. The orange box is the possible pathogenic gene area.

Evaluation of the chromosomes from peripheral blood lymphocytes revealed a 46, XY karyotype in all the metaphase cells. But the clinical microarray analysis revealed a 10Mb deletion in the short arm of chromosome 1 with breakpoints in 1p33 and 1p32.2 (chr1: 47831383–58364913) (Fig. 2). The deleted region includes the *ORC1*, *DAB1* and *SCP2* gene, amongst the 44 OMIM gene. There was an overlapped deletion region with 1p32-p31 deletion syndrome whose critical gene was *NFIA* (Fig. 3); another overlapped deletion region with 1p32 deletion was also found (Fig. 4).

We compared the clinical features of our patient to the ones who carried the Chromosome 1p33p32.2 deletion from the Decipher database^[11]^ (Table 1). DD/ID was observed in most of the patients with partial 1p deletion. Our patient exhibited many features that are not frequently observed in other 1p cases such as Pierre Robin sequence, special craniofacial dysmorphisms (microcephaly, dental crowding, frontal bossing and prominent philtrum), delayed speech and language development, growth retardation and short stature. Our patient also presented with some physical features that had not been described in other case of 1p deletion such as high palate, sparse eyebrows, long face, squint, and nystagmus. In addition to the physical features previously noted, some other specific features also have been observed in this patient such as myelination abnormality of white matter (leukoencephalopathy), aggression, seizure, and food allergy.

**Table 1:** Phenotype comparison of patient in the present and the previously reported ones.

	Decipher ID (patient)								
	
Variables	4638^[11]^	272313^[11]^	262636^[11]^	276510^[11]^	274571^[11]^	251738^[11]^	Mulatinho et al. 2008 (Mulatinho, 2008 #24)^[17]^	Kehrer et al. 2015 (Kehrer, 2015 #18)^[18]^	Our case
Sex and age	Female, 13 yr	Female, 6 yr	Female, 14 yr	Male, 3 yr	Female, 3 yr	Female, 8 yr	Male, 25 yr	Male, 17 mo	Male, 3 yr
Size (Mb)	24.59	7.3	7.3	4.58	3.54	0.69	5.4	6.4	10.53
Inheritance	Unknown	De novo	Unknown	De novo	Unknown	De novo	De novo	De novo	De novo
Phenotype	NA	NA	NA	NA	NA	NA	NA	NA	NA
Micrognathia	NA	+	+	NA	NA	NA	_	+	+
High palate	NA	NA	NA	NA	NA	NA	NA	NA	+
Dental crowding	NA	NA	NA	NA	NA	NA	+	NA	+
Sparse eyebrows	NA	NA	NA	NA	NA	NA	NA	NA	+
Frontal bossing	NA	+	NA	NA	NA	NA	_	_	+
Long face	NA	NA	NA	NA	NA	NA	_	_	+
Prominent philtrum	NA	NA	NA	+	NA	NA	+	_	+
Squint	NA	NA	NA	NA	NA	NA	NA	NA	+
Nystagmus	NA	NA	NA	NA	NA	NA	NA	NA	+
Microcephaly	NA	+	+	NA	NA	NA	_	+	+
Short stature	NA	NA	NA	NA	NA	NA	+	_	+
Developmental delay/ID	NA	+	+	+	+	+	+	NA	+
Feeding difficulties in infancy	NA	NA	NA	NA	NA	+	NA	NA	+
Myelination delay of white matter	+	NA	NA	NA	NA	NA	_	_	+
Aggression	NA	NA	NA	NA	NA	NA	NA	NA	+
Seizure	NA	NA	NA	NA	NA	NA	NA		+
Food intolerance	NA	NA	NA	NA	NA	NA	NA	NA	+
Delayed speech and language development	NA	NA	NA	+	NA	NA	+	+	+
Growth retardation	NA	NA	NA	NA	NA	NA	_	_	+

After the genetic diagnosis, the patient received language and cognitive rehabilitation training in the special rehabilitation facilities. The intermittent rehabilitation training lasted for 1 year and the child's language expression and learning ability improved. However, due to economic reasons, he stopped the training. The child is currently in the first grade of elementary school where his language and academic performance is below average.

This study was approved by the Ethics Committee of children's Medical Center affiliated to Shanghai Jiao Tong University School of Medicine (SCMCIRB-K2014053).

## 3 Discussion

This paper presents a case of a 39-month boy with Pierre Robin sequence, DD/ID, growth retardation, short stature, leukoencephalopathy, craniofacial dysplasia, and speech delay. CMA detected a 10 Mb microdeletion at 1p33p32.2. Three candidate genes were pinpointed to the deleted area, including *ORC1, SCP2,* and *DAB1*. This case could represent a new microdeletion syndrome.

Karyotype analysis usually has good resolution in clarifying 5 to 10Mb chromosome aberrations; however, accurate results depend upon the laboratory technician's experience and skills. As in our patient, a 10 Mb deletion was missed by G-band karyotype analysis, but it was subsequently identified by CMA analysis. This suggests that CMA has better resolution and sensitivity in diagnosis, and it is a good back up for G-band karyotype analysis.

Extensive database research (OMIM, PubMed, and Decipher) revealed no other case study with 1p33p32.2 deletion at the 1p locus, and this is the largest one involving partial 1p deletion. The partial 1p deletion in this patient is 10Mb in size, harbors 44 OMIM genes, 9 of which are disease-causing, including: *BSND* (OMIM:606412), *C8A* (OMIM:120950), *C8B* (OMIM:120960), *PCSK9* (OMIM:607786), *CPT2* (OMIM:600650), *LRP8* (OMIM:602600), *DHCR24* (OMIM:606418), *SCP2* (OMIM:184755), and *ORC1* (OMIM:601902). Mutations in *BSND* can cause either autosomal recessive sensorineural deafness with only mild renal problems or Bartter syndrome type 4a (OMIM:602522).^[12]^ Deficiency of *C8A* and *C8B* causes recurrent Neisseria infections, predominantly with meningococcus infection of rare serotypes which is not present in our patient.^[13]^ Interestingly, some of the genes are known to be involved in fatty acid oxidation and metabolism including *CPT2; LRP8; PCSK9;* and *DHCR24*, whose deletion results in abnormal lipid metabolism. Cholesterol serum levels were, however, normal in our patient. Mutation in *ORC1* can cause growth retardation, microcephaly, and short stature (OMIM:224690),^[14]^ which can explain part of the phenotype of the case. Mutation in *SCP2* can cause leukoencephalopathy with dystonia and motor neuropathy and accumulation of the branched-chain fatty acid pristanic acid in plasma (OMIM:613724).^[15]^ Seedorf et al. demonstrated that mice with targeted *SCP2* gene disruption developed ataxia, reduced muscle tone, and peripheral neuropathy (uncoordinated movements, unsteady gait, and trembling).^[16]^ As expected in this case of *SCP2* haploinsufficiency, leukoencephalopathy was present, which indicates that *SCP2* may explain part of the phenotype of our case. Motor neuropathy and dystonia was not shown at 39 months of age, but considering the size of the deletion and the deficits shown in the other patients with deletion of *SCP2*, future neurologic disorder are conceivable.

Some academics have reported on 1p31.3p32.2 deletion syndrome and 1p32 deletion and these cases have partially overlapping deletion regions with our case.^[17–19]^ However, they have specific clinical feature, and the deletion regions have different sizes and positions. Compared with 1p31.3p32.2 deletion syndrome, there was an obvious overlapping deletion region-1p32.2 (Fig. 2), and some similar phenotypes were also observed, such as developmental delay, craniofacial dysmorphia and central nervous system malformation.^[20]^ But the critical region of 1p31.3p32.2 deletion syndrome is the locus on 1p31.3 involving the *NFIA* gene which is responsible for all the phenotypes such as craniofacial dysplasia, hypoplasia of the corpus callosum, developmental delay, metopic synostosis and urinary tract abnormalities.^[18]^ As *NFIA* is not included in the CNV region of our case, we suppose that some other gene(s) may be responsible for our case's phenotypes. Multinho et al^[17]^ have reported a de novo, 5.4 Mb interstitial deletion at 1p32.2p32.3 with intellectual disability, low level of total and LDL cholesterol, short stature, speech delay, and dysmorphism. Similar phenotypes to our case report were described, such as short stature, intellectual disability, speech delay, prominent philtrum, and dental crowding. However, a large overlapping deletion region was also found in that case, in contrast with ours (Fig. 3). It should be noted that the chromosomal band 1p32.2, and the *PCSK9*, *DAB1,* and *SCP2* genes, was affected in both cases. Another interstitial deletion at 1p32.1p32.3, 6.4 Mb in size, was reported by Kehrer et al^[21]^ who described a 17-month-old boy with features of low LDL cholesterol, choanal atresia, delayed speech and language development, hearing loss, urogenital anomalies, and craniofacial dysmorphism (microcephaly, flat nasal bridge, small nose, anteverted nares, retrognathia), also effected the *PCSK9* and *DAB1* genes, with few similar clinical features with our case. Mutations in *PCSK9* cause autosomal dominant cholesterol metabolism disorder (OMIM 603776), the clinical features of which include hypocholesterolemia and hypercholesterolemia.^[22]^ While, cholesterol serum levels were normal in our patient. The brain-expressed gene *DAB1*, which is disrupted in our case, encodes for an obligate effector of the Reel in signaling pathway with two other cell-surface receptors, *VLDLR* (OMIM: 192977) and *LRP8* (also known as Apolipoprotein E receptor-2; OMIM: 107741). *DAB1* is critical for neuronal migration and dendrite outgrowth during development,^[23]^ and is a plausible candidate gene responsible for our case's global DD/ID.

In our patient, Pierre Robin sequence was initially suspected when he was born. Pierre Robin sequence is not a syndrome itself, but rather a congenital malformation which is characterized by micrognathia, glossoptosis and upper airway obstruction with or without cleft palate which is usually compatible with syndromic diagnosis, such as for Stickler syndrome, velocardio-facial syndrome, and Treacher-Collins syndrome.^[24,25]^ Obviously, our case belongs to none of these syndromes. *SOX9, KCNJ2, Ptprs* and *Ptprf* are probably connected with Pierre Robin sequence, but research on those genes has focused on 17q23–24, whose mutations are not detected in our case.

This study has some limitations. This is a case report of just one individual, so it is very difficult to provide definite evidence of candidate genes for the syndrome. Additional experiments and laboratory techniques are needed to narrow down the candidate genes that correlate to all of the phenotypes in this case, and more patients with chromosome aberrations in genes of this region need to be identified to establish better genotype-phenotype correlations and to clarify the role of individual genes for the multiple clinical manifestations. However, as this is a very rare case the data presented should provide important information on the approach to diagnosis of similar cases.

Our study gives a comprehensive description of a 10 MB 1p33p32.2 deletion with a significant phenotype. Three genes in the region: *ORC1, SCP2, DAB1*, may be candidates for the main phenotypes observed in our patient such as short stature, microcephaly, growth retardation, leukoencephalopathy and DD/ID. Further study is needed to explain the other phenotypes, such as craniofacial abnormality, delayed speech and language development, Pierre Robin sequence, and seizure.

## Acknowledgments

We gratefully acknowledge the contribution of all participants, supporting staff, and referring physicians to this project.

## Author contributions

**Conceptualization:** Fei Li.

**Investigation:** Maoying Jiang, Shanlin Wang, Juan Geng, Yiting Ji, Ke Li, Xiaodong Jiang.

**Methodology:** Maoying Jiang, Shanlin Wang, Juan Geng, Yiting Ji, Ke Li, Xiaodong Jiang.

**Writing – original draft:** Maoying Jiang.

**Writing – review & editing:** Fei Li.
